# Retraction: Baicalin suppresses lung cancer growth by targeting PDZ-binding kinase/T-LAK cell-originated protein kinase

**DOI:** 10.1042/BSR20181692_RET

**Published:** 2026-10-14

**Authors:** Xin Diao, Danfen Yang, Yu Chen, Wentian Liu

**Affiliations:** 1 Department of Pulmonary and Critical Care Medicine, The First Affiliated Hospital of Xi'an Medical University, Xi'an 710077, China; 2 Department of Cadre Ward, Affiliated Hospital of Yan'an University, Yan'an 716000, China; 3 Department of Extracoracic Oncology, Baoji People's Hospital, Baoji 721000, China; 4 Department of Respiratory Medicine Hanzhong Central Hospital, Hanzhong 723000, China

**Keywords:** Baicalin, inhibitor, lung cancer, PBK/TOPK

This article is being retracted from *Bioscience Reports* at the request of the Editor-in-Chief and the Editorial Board. This follows the receipt of a notification from a reader, alerting the Editorial Board to a number of similarities between the manuscript and a paper published in another journal by different authors. Upon investigation, the Editorial Office identified a number of additional concerns. Similarities noted between images are described below:

- Figure 2C's graph and the Figure 2C graph from Xingyu et al. 2016 (doi: 10.1002/cam4.89)
- Figure 3E p-Histone H3 panel and the Figure 2B Active Caspase 3 panel from Cai et al. 2021 (doi: 10.3389/fphar.2021.650193)
- Figure 3E p-Histone H3 panel and the Figure 5A p-NF-kB p65 panel from Fan et al. 2016 (doi: 10.18632/oncotarget.8260)
- Figure 5B images and Figure 4B images from Fan et al. 2019 (doi: 10.3389/fphar.2019.01248)
- Figure 5C Histone H3 panel and the Figure 4D STK33 panel from Zhang et al 2019 (doi: 10.1042/BSR20182351) following 180° rotation
- Figure 5C Histone H3 panel and the Figure 3B p38 panel from Fan et al. 2016 following a vertical flip (doi: 10.18632/oncotarget.8260)
- Figure 6C p-Histone H3 vehicle control panel and the Figure 6C Untreated p-H3 panel from Yang et al. 2016 (doi: 10.1016/j.jgr.2016.03.007)

The authors have been contacted with regard to the retraction and have not responded to the Journal's queries or the concerns.

Given the extent of the issues raised, the Editorial Board stands by the decision to retract the article.

